# Making the most of approximate maximum common substructure search

**DOI:** 10.1186/1758-2946-6-S1-P29

**Published:** 2014-03-11

**Authors:** Péter Englert, Péter Kovács

**Affiliations:** 1 Department of Algorithms and Applications, Eötvös Loránd University, Budapest, H-1117, Hungary; 2 ChemAxon Ltd., Budapest, H-1031, Hungary

## 

The maximum common substructure (MCS) problem is of great importance in multiple aspects of chemoinformatics. It has diverse applications ranging from lead prediction to automated reaction mapping and visual alignment of similar compounds. Many different algorithms have been developed [1], both exact and approximate. Since the MCS problem is NP-complete, the strict time constraints of most applications can only be realistically satisfied by fast and robust approximation methods.

We developed two efficient heuristic algorithms. One is based on the popular approach of reducing the MCS problem to finding the maximum clique in the modular product of the two molecule graphs. The other is based on a new algorithm by Kawabata, called the build-up method [2]. We also incorporated other techniques, for example, the topological fingerprinting primarily used in substructure and similarity searching. We optimized our implementations for use in multiple applications developed at ChemAxon. In some applications, for example, hierarchical MCS-based clustering or similarity search in large databases, the algorithms are required to give close to optimal results in limited time. To meet these conflicting demands, our implementations were enhanced with strong heuristics. Upper bound calculation methods were also applied for screening and early termination purposes.

In other applications, for example, reaction mapping or visual alignment, the challenge is that topological features must also be taken into account. Apart from the size of the found common substructure, the determined one-to-one correspondence between the atoms of the molecules is also very important. Effective heuristics were developed to guide the algorithms to prefer those solutions in which the relative positions of the common fragments of the input molecules are as similar as possible.

Our implementations have been thoroughly tested and benchmarked. They have also been compared to publicly available solution methods, and integrated into different products at ChemAxon. This has shown that the presented MCS algorithms can adequately cover the conflicting requirements of typical applications. We present our methods and heuristics along with their effects on running time, memory usage, as well as the size and features of the result.
